# Clinicopathological and Molecular Profiles of Sporadic Microsatellite Unstable Colorectal Cancer with or without the CpG Island Methylator Phenotype (CIMP)

**DOI:** 10.3390/cancers12113487

**Published:** 2020-11-23

**Authors:** Shih-Ching Chang, Anna Fen-Yau Li, Pei-Ching Lin, Chun-Chi Lin, Hung-Hsin Lin, Shen-Chieh Huang, Chien-Hsing Lin, Wen-Yi Liang, Wei-Shone Chen, Jeng-Kai Jiang, Jen-Kou Lin, Shung-Haur Yang, Yuan-Tzu Lan

**Affiliations:** 1 Division of Colon & Rectal Surgery, Department of Surgery, Taipei Veterans General Hospital, Taipei 11217, Taiwan; changsc@vghtpe.gov.tw (S.-C.C.); cclin15@vghtpe.gov.tw (C.-C.L.); hhlin7@vghtpe.gov.tw (H.-H.L.); schuang5@vghtpe.gov.tw (S.-C.H.); wschen@vghtpe.gov.tw (W.-S.C.); jkjiang@vghtpe.gov.tw (J.-K.J.); jklin@vghtpe.gov.tw (J.-K.L.); yangsh@vghtpe.gov.tw (S.-H.Y.); 2 Department of Surgery, Faculty of Medicine, School of Medicine, National Yang-Ming University, Taipei 11121, Taiwan; 3 Department of Pathology, Taipei Veterans General Hospital, Taipei 11217, Taiwan; fyli@vghtpe.gov.tw (A.F.-Y.L.); wyliang@vghtpe.gov.tw (W.-Y.L.); 4 Department of Clinical Pathology, Yang-Ming Branch, Taipei City Hospital, Taipei 11146, Taiwan; b7901127@tmu.edu.tw; 5 Department of Health and Welfare, University of Taipei, Taipei 11153, Taiwan; 6 Division of Genomic Medicine, National Health Research Institutes, Zhunan 35053, Taiwan; jameslin@fcbiotech.com.tw

**Keywords:** colorectal cancer, microsatellite instability, mutation, CpG island methylator phenotype

## Abstract

**Simple Summary:**

The 5’-C-phosphate-G-3’ island methylator phenotype (CIMP) is a phenotype of colorectal cancer associated with microsatellite instability (MSI). The aim of the present study was to analyze the CIMP phenotype and genetic mutations in MSI-high colorectal cancer. Our results demonstrated that among MSI-high colorectal cancer patients, CIMP-high tumors were associated with specific mutation profiles and clinicopahtological features compared with CIMP-low or CIMP-0 tumors, however, the CIMP status was not an independent prognostic factor.

**Abstract:**

Background: The 5’-C-phosphate-G-3’ island methylator phenotype (CIMP) is a specific phenotype of colorectal cancer (CRC) associated with microsatellite instability-high (MSI-high) tumors. Methods: In this study, we determined the CIMP status using eight methylation markers in 92 MSI-high CRC patients after excluding five germline mismatch repair (MMR) gene mutations analyzed by next-generation sequencing (NGS) and confirmed by Sanger sequencing. The mutation spectra of 22 common CRC-associated genes were analyzed by NGS. Results: Of the 92 sporadic MSI-high tumors, 23 (25%) were considered CIMP-high (expressed more than 5 of 8 markers). CIMP-high tumors showed proximal colon preponderance and female predominance. The mutation profiles of CIMP-high tumors were significantly different from those of CIMP-low or CIMP-0 tumors (i.e., higher frequencies of *BRAF*, *POLD1*, *MSH3*, and *SMAD4* mutations but lower frequencies of *APC*, *TP53*, and *KRAS* mutations). Multivariate analysis demonstrated that tumor, node, metastasis (TNM) stage was the independent prognostic factor affecting overall survival (OS). Among the MSI-high cases, the CIMP status did not impact the outcome of patients with MSI-high tumors. Conclusions: Only TNM stage was a statistically significant predictor of outcomes independent of CIMP profiles in MSI-high CRC patients. Sporadic MSI-high CRCs with different mechanisms of carcinogenesis have specific mutation profiles and clinicopathological features.

## 1. Introduction

Approximately 10–15% of colorectal cancers (CRCs) have a high frequency of microsatellite instability (MSI) [1,2,3]. MSI-high CRC showed a proximal colon predominance, a female predominance, and a more mucinous histology than microsatellite-stable (MSS) CRC [4,5,6]. The majority of MSI-high CRCs are believed to originate from defects in or the loss of expression of DNA mismatch repair (MMR) proteins, including MLH1, MSH2, MSH6, PMS2, and EPCAM [7,8,9]. A deficiency in MMR genes results in the inefficient correction of errors in repetitive sequences (microsatellite DNA) during DNA synthesis [10,11].

Germline mutations in a DNA MMR gene are observed in ninety percent of CRC patients with Lynch syndrome (LS), and these patients have the MSI-high phenotype, the most frequent cause of hereditary CRC, with a frequency of 1–3% of CRCs [1,2]. Germline mutations in MMR genes, *MLH1* silencing due to hypermethylation of the promoter of *MLH1*, or somatic mutations in MMR genes in tumor cells are the other causes of MSI [8,9,10].

A subset of CRCs (up to 15%) are associated with the 5’-C-phosphate-G-3’ island methylator phenotype (CIMP), which includes *MLH1* hypermethylation [11,12,13]. The clinicopathological features of CIMP-positive CRCs are unique and overlap with those of MSI-high CRCs [14,15]. Studies have demonstrated that the rate of *BRAF* mutations is higher in MSI-high CRCs than in MSS CRCs, especially those associated with *MLH1* hypermethylation [16,17]. Because the *BRAF* mutation is found exclusively in individuals without LS [18,19,20], it can be used as a surrogate marker to differentiate LS from sporadic MSI-high CRC. The mechanisms of carcinogenesis and molecular profiles of LS have been well studied. However, the molecular profiles of sporadic MSI-high CRC with/without CIMP positivity are not well understood.

In gene promoter regions, there are many 5’-C-phosphate-G-3’ (CpG) dinucleotides, also called CpG islands. Methylation usually occurs at the cytosine bases of CpG dinucleotides, and promoter methylation can regulate gene expression [21]. The dysregulation of methylation increases in response to chronic inflammation, other stimuli, aging, etc. [22,23,24]. The hypermethylation of promoter regions can result in the silencing of tumor suppressor genes and induce the carcinogenesis of many cancers, including CRC [21,22,23,24,25]. The definition of CIMP-positive tumors is inconclusive. Most studies have applied one of two panels: the Ogino panel and the Weisenberger panel [14,17]. The advantage of the Ogino panel is that this panel consists of eight markers, including five markers used in the Weisenberger panel.

In this study, we selected 92 sporadic MSI-high CRCs after excluding germline mutations in five MMR genes: *EPCAM*, *MLH1*, *MSH2*, *MSH6*, and *PMS2.* According to the Ogino panel [4], we identified twenty-three (25%) CIMP-high tumors as having at least five positive markers with hypermethylation. Furthermore, we compared the difference in somatic mutations in five MMR genes and another 17 common genes associated with CRC analyzed by next-generation sequencing (NGS) between CIMP-high, CIMP-low, and CIMP-0 tumors.

## 2. Results

### 2.1. Clinical Data 

As shown in Table 1, of the 92 patients with MSI-high CRC, 43 were females (46.7%) The mean age at the time of tumor resection was 69.0 ± 14.6 (median: 71.5) years. The histological examination showed 11 (12.0%) patients with stage I, 48 (52.2%) with stage II, 20 (21.8%) with stage III, and 13 (14.1%) with stage IV disease as defined by the TNM staging system. LVI, mucinous histology, and poor differentiation were present in 14 (15.2%), 15 (16.3%), and 13 (14.1%) patients, respectively.

### 2.2. Alterations in Molecular Profiles

The case numbers and frequencies of hypermethylation markers were as follows: *CRABP1* (44, 47.8%); *CDKN2A* (40, 43.5%); *CACNA1G* (36, 39.1%); *IGF2* (31, 33.7%); *RUNX3* (30, 32.6%); *MLH1* (26, 28.3%); *NEUROG1* (18, 19.6%); and *SOCS1* (16, 17.4%). Twenty-three (25%) CRC were considered CIMP-high. The case numbers and frequencies of somatic mutations in five MMR genes were as follows: *MSH6* (41, 44.6%), *MLH1* (20, 21.7%), *PMS2* (19, 20.7%), *MSH2* (16, 17.4%), and *EPCAM* (6, 7.5%). Twenty-eight patients with sporadic MSI-high CRC (30.4%) did not have somatic mutations in these five MMR genes. Twenty-two patients (23.9%) had neither CIMP-positive nor MMR mutations. The case numbers and frequencies of 17 common CRC-associated genes are shown in Table 2. In sporadic MSI-high CRC, the most common mutated genes (more than 40%) were *PMS1* (76, 82.7%), *BAX* (48, 52.2%), *KRAS* (45, 48.9%), *POLE* (44, 47.8%), *TP53* (41, 44.6%), and *APC* (40, 43.4%).

### 2.3. Clinicopathological Features and Molecular Signatures of Microsatellite Instability (MSI)-High Colorectal Cancer (CRC) Classified by the 5′-C-phosphate-G-3′ Island Methylator Phenotype (CIMP) Status

The relationships between the CIMP and clinicopathological features are shown in Table 1. Patients with sporadic MSI-high and CIMP-high CRCs showed a female predominance (73.9%), a proximal tumor location predominance (82.6%), and poorer differentiation (34.8%) than those with CIMP-low or CIMP-0 CRCs. 

**Figure 1:**
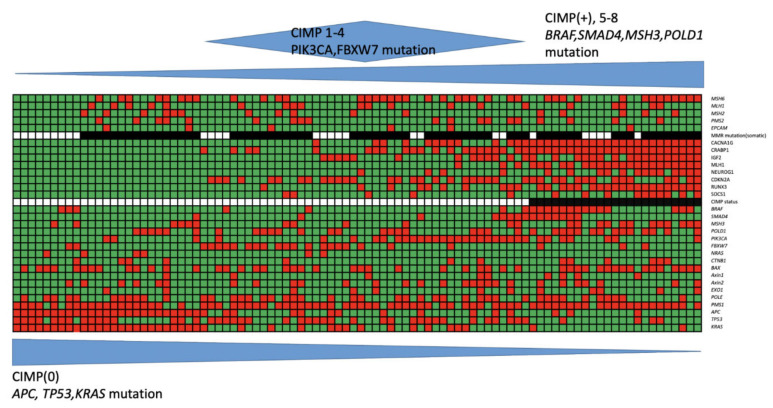
The mutational profiles in microsatellite instability (MSI)-high colorectal cancer (CRC) with 5′-C-phosphate-G-3′ island methylator phenotype CIMP-high, CIMP-low, and CIMP-0 CRCs. Green field: genetic mutation (−); red field: genetic mutation (+); white field: MMR mutation (−) or CIMP−; black field: MMR mutation (+) or CIMP+; blue field: rate of genetic mutation.

Table 2 and Figure 1 show the different mutation frequencies between CIMP-high, CIMP-low, and CIMP-0 CRCs with MSI-high tumors. CIMP-high tumors had a significantly lower frequency of mutations in *APC* (13%), *TP53* (8.7%), and *KRAS* (17.4%) than CIMP-low or CIMP-0 tumors. In contrast, CIMP-high tumors had a significantly higher frequency of mutations in *BRAF* (60.9%), *POLD1* (60.9%), *MSH3* (52.2%), and *SMAD4* (30.4%) than CIMP-low or CIMP-0 tumors. The somatic mutations in five major MMR genes were distributed similarly among CIMP-high, CIMP-low, and CIMP-0 tumors (73.9% vs. 70.5% vs. 64.0%, *p* = 0.745).

As shown in Table S1, CIMP-high tumors had a higher frequency of loss of MMR protein expression than CIMP-low or CIMP-0 tumors (73.9% vs. 38.6% vs. 32.0%, *p* = 0.006), especially in *MLH1* (56.5% vs. 22.7% vs. 12.0%, *p* = 0.002) and *PMS2* (60.9% vs. 38.6% vs. 24.0%, *p* = 0.032).

### 2.4. Clinicopathological Features and Molecular Signatures of MSI-High CRC Classified by Somatic Mismatch Repair (MMR) Mutations

The correlations between clinicopathological features and somatic MMR mutations are shown in Table 3. Mucinous histology was found in 20.3% of MSI-high tumors with MMR mutations; this rate was significantly higher than that in MSI-high tumors without MMR mutations (0%, *p* = 0.008). The other clinicopathological features were similar between these two groups of tumors. As shown in Table 4, the frequency of *POLD1* mutations was significantly higher in MSI-high tumors with somatic MMR mutations than in those without MMR mutations (46.9% vs. 21.4%, *p* = 0.036).

### 2.5. The Mutational Profiles in MSI-High CRC Classified by the Cause of MSI

**Figure 2:**
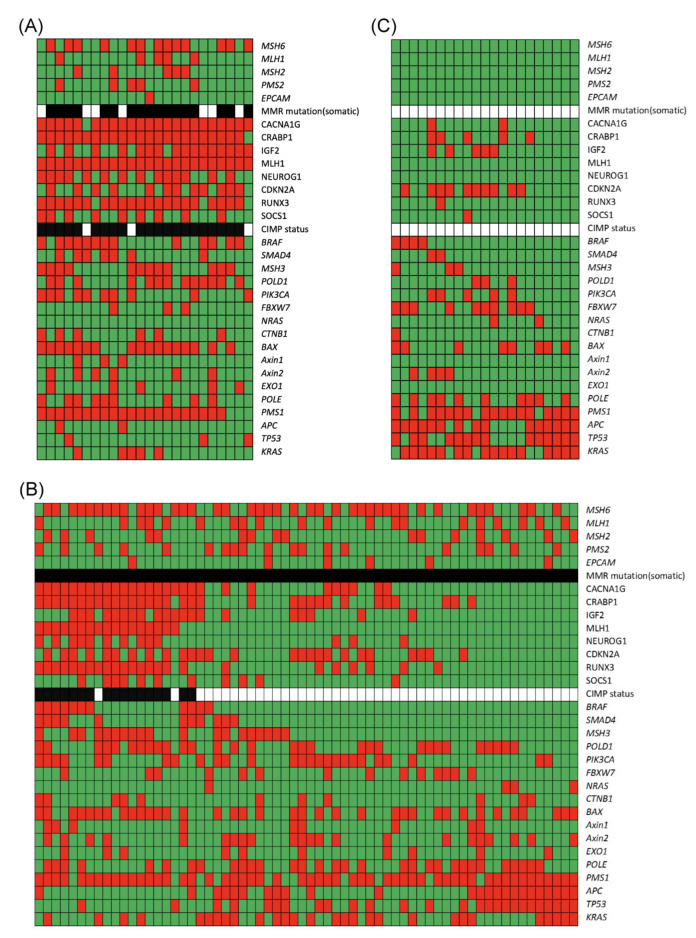
The mutational profiles in MSI-high CRC according to the cause of MSI-high. (**A**) MSI-high CRC with *MLH1* methylation, (**B**) MSI-high CRC with MMR mutation, (**C**) MSI-high CRC with no obvious cause for MSI-high. Green field: genetic mutation (−); red field: genetic mutation (+); white field: no MMR mutation or CIMP−; black field: MMR mutation or CIMP+.

As shown in Figure 2A, for MSI-high CRC with *MLH1* methylation (*n* = 24), the most common mutated MMR gene was *MSH6*, followed by *MLH1* and *MSH2*. Most of the patients (87.5%, 21/24) had CIMP-high tumors. The most common mutated gene was *PMS1*, followed by *BAX*.

As shown in Figure 2B, for MSI-high CRC with an MMR mutation (*n* = 64), the most common mutated MMR gene was *MSH6*, followed by *MLH1*. Among the 64 patients, 17 (26.6%) had CIMP-high tumors. The most common mutated gene was *PMS1*.

As shown in Figure 2C, for MSI-high CRC with no obvious cause of MSI-high (*n* = 21), there were no patients with an MMR mutation or CIMP-high tumors. The most common mutated gene was *PMS1*, followed by *KRAS*, *APC*, and *TP53*.

### 2.6. Impact of the CIMP and Somatic MMR Statuses on Outcomes of Colorectal Cancer Patients

The 5-year DFS rate of 23 patients with CIMP-high CRC was slightly better than that of 69 patients with CIMP-low or CIMP-0 CRC, without statistical significance (79.9% vs. 52.5%, *p* = 0.158). The CIMP status did not impact the 5-year OS rate of CRC patients with MSI-high tumors (61.4% vs. 59.3%, *p* = 0.921). Patients with somatic MMR mutations and those without MMR mutations had similar 5-year DFS and OS rates.

As shown in Table 5, the univariate analysis demonstrated that TNM stage, LVI, and adjuvant chemotherapy were significantly associated with OS in these 92 patients with MSI-high CRC. The aforementioned three covariates were included in the multivariate analysis. Multivariate Cox regression analysis demonstrated that TNM stage was the independent prognosticator for patients with MSI-high CRC. Neither CIMP nor the somatic MMR status impacted the OS of patients with MSI-high CRC.

## 3. Discussion 

Our results address three major issues regarding sporadic MSI-high CRC. First, 25% of sporadic MSI-high CRCs were associated with aberrant DNA methylation; second, the molecular and clinicopathologic features of CIMP-high CRCs were specific to sporadic MSI-high CRCs; third, the molecular and clinicopathological features of sporadic MSI tumors with and without MMR mutations were similar.

Because the patients enrolled in the present study were diagnosed between 2000 and 2010, the original MSI definition by the National Cancer Institute from 1998 was used [4]. The definition of MSI-H was updated in 2008, with at least one mononucleotide repeat marker needing to be mutated to warrant an MSI-H classification [16]. Using the original MSI definition rather than the version updated in 2008, a substantial proportion of the MSI-H tumors without a somatic MMR gene mutation or *MLH1* methylation may actually reflect a low level of MSI, which might have impacted our data.

Regarding the CIMP status, most studies have one of two panels: the Ogino panel [14] or the Weisenberger panel [17]. There are eight markers used in the Ogino panel, and the rate of CIMP-high in sporadic MSI-H CRC was 72.7% (88/121), whereas the rate of *BRAF* mutation was 45% (53/118); the definition of the MSI phenotype in the Ogino study [26] using 10 markers defined MSI-H as the presence of instability in ≥30% of the markers. In contrast, the Weisenberger panel consists of five markers, all of which are included in the eight markers of the Ogino panel, and the rate of CIMP-high in sporadic MSI-H CRC was 57.1% (12/21). The definition of the MSI phenotype in the Weisenberger study consisted of five markers: two mononucleotides (*BAT25*, *BAT26*) and three dinucleotides (*D5S346*, *D2S123*, and *D17S250*) [4]. The CIMP used in the present study was the same as that used in the study by Ogino et al. [14], while the MSI phenotype used in the present study was the same as that used in the study by Weisenberger et al. [17].

In the general population of CRC patients, the CIMP-high rate varies widely and ranges from 6.4–48% [11,12,13,14,15,16,17,27,28,29,30]. Ogino’s study demonstrated that the frequency of CIMP-high tumors varied depending on the cutoffs and genes included in a panel [31]. The present study followed Ogino’s definition, including the cutoff value of hypermethylation. Because we selected samples from sporadic MSI-high tumors, it was reasonable that 25% of tumors were considered CIMP-high. In the present study, among MSI-H CRC tumors, the rate of CIMP-high was 25% and the rate of *BRAF* mutation was 22.8%, which was lower than that in the study by Ogino et al. among a Western population (72.7% CIMP-H and 45% *BRAF* mutation) [14]. The discrepancies between studies might be due to differences in the definitions of CIMP and MSI or in the patient population, ethnicities, or environmental and genetic factors.

All eight markers used in the Ogino panel showed sensitivities of >60% and specificities of ≥80% for CIMP+ tumors [31]. The Ogino panel with eight markers was more accurate in reflecting the true CIMP status than any other marker combination: thus, the eight-marker panel has been designated as the gold standard [31]. In addition, regarding the *BRAF* and *KRAS* mutation frequencies, the authors demonstrated that the Ogino panel with eight markers was a superior gold-standard panel (44% *KRAS*-mutated, and 33% *BRAF*-mutated) to the Weisenberger panel with five markers (43% *KRAS*-mutated, and 28% *BRAF*-mutated) [31]. Ogino et al. stated that all eight markers are good surrogate markers for determining the CIMP status, and at least four markers, namely, *RUNX3*, *CACNA1G*, *IGF2*, and *MLH1* constitute a sensitive and specific CIMP panel [31]. In contrast, the five markers used in the Weisenberger panel are *CACNA1G*, *IGF2*, *NEUROG1*, *RUNX3*, and *SOCS1*, and the panel does not include *MLH1*. Despite of the facts mentioned above, the Weisenberger panel is used more often than the Ogino panel. The reason might be that it is easier to analyze the CIMP status using fewer markers. However, investigating the CIMP status according to the different panels among different races as well as their clinical relevance is necessary.

Regarding the Ogino panel, the percentage of CIMP was 71.9% (92/128) among MSI-H tumors with a PMR cutoff of 4, which was decreased to 15.6% (20/128) with a PMR cutoff of 6 [31]. In the present study, among the 92 MSI-H CRCs, the percentage of CIMP was 25% (23/92) with a PMR cutoff of 4, which was decreased to 9.8% (9/92) with a PMR cutoff of 6. As a result, a PMR cutoff of 4 was used in the present study to increase the number of CIMP-high patients.

An earlier study concluded that most sporadic MSI-high CRCs originated from hypermethylation of the *MLH1* gene promoter [32]. In vitro, the *MLH1* promoter was demethylated in MSI-high CRC cell lines, providing evidence that the number of MSI events decreases as *MLH1* is reactivated (i.e., in expression and function) [33,34]. Therefore, aberrant DNA methylation is considered the cause but not the consequence of sporadic MSI-high CRC. However, our results showed that only 26.1% (24/92) and 25% of sporadic MSI-high tumors had *MLH1* hypermethylation and were considered CIMP-high, respectively. Another mechanism, such as somatic mutations in MMR genes, was demonstrated in several studies to be a possible cause of sporadic MSI-high CRC [5,6,35]. Our results demonstrated that most sporadic MSI-high CRCs originated from somatic MMR mutations (69.6%). Seventeen patients (18.5%) had both CIMP-high tumors and somatic MMR mutations. Compatible with previous studies showing that somatic mutations in MMR genes were frequent causes of MMR deficiency in LS-like tumors [5,6,34], half of the sporadic MSI-high tumors in our series (46/92) had somatic mutations in MMR genes after exclusion of the CIMP-positive status or *MLH1* hypermethylation. In total, seventy tumors (76.1%) were classified as MSI-high. MSI-high tumors with and without somatic MMR mutations had similar clinicopathological features. The mutation rates of other genes were also similar between MSI-high tumors with and without somatic mutations. Patient outcomes were also similar between the two groups. These results indicate that MSI determines the tumor signatures but not the origin of mutations.

It was reported that CIMP-high CRC was strongly associated with MLH1 hypermethylation and BRAF mutations [30], which was similar to our results as shown in Figure 2A,B. For CIMP-high CRC, MLH1 hypermethylation was associated with MSI-H status [36]. It seems that MLH1 methylation plays an important role in CIMP-high CRC with MSI-H.

Our results demonstrated that CIMP-high tumors were more likely to lose expression of MMR protein, especially *MLH1* and *PMS2*. In addition, among the 24 CRC patients with *MLH1* hypermethylation, 16 patients (66.7%) had loss of expression of *MLH1*. In the present study, all of the 26 patients (100%) with loss of expression of *MLH1* had loss of expression of *PMS2*. The heterodimeric nature of the MMR proteins is possibly the reason to explain our findings. Loss of expression of one MMR protein may be due to the loss of expression of its paired protein [37].

In the present study, 44 (47.8%) tumors were CIMP-low and 25 (27.2%) were CIMP-0. As shown in Figure 1, all of the CIMP-0 tumors had *APC* (100%) and *TP53* (100%) mutations, and the mutation frequency decreased as the number of methylation markers decreased. The meta-analysis also showed that the CIMP-high status was inversely associated with the *TP53* mutation [38]. In addition, the mutation frequency of *KRAS* reached up to 60% in CIMP-0 and CIMP-low tumors but only 17.4% in CIMP-high tumors. 

Interestingly, we found that CIMP-low tumors had a significantly higher mutation rate in *FBXW7* than CIMP-high and CIMP-0 tumors. Nearly 50% of CIMP-low tumors had the *FBXW7* mutation. As shown in Figure 1, the majority of tumors with the *FBXW7* mutation had fewer than three hypermethylation markers. To date, no report has mentioned the association between the *FBXW7* mutation and aberrant DNA methylation. In The Cancer Genome Atlas (TCGA) study, the majority of MSI-high CRCs with silenced *MLH1* expression had few *APC*, *KRAS*, *TP53*, and *FBXW7* mutations [29]. Another validation study showed that 50% of non-*MLH1*-methylated MSI-high CRCs contained truncating *APC* mutations [39]. Our results combined with previous results suggest that sporadic MSI-high CRCs with a low level of aberrant DNA methylation have a relatively higher mutation rate of proteins involved in canonical pathways, including *APC*, *KRAS*, *TP53*, *and FBXW7*, than those with a high level of aberrant DNA methylation [40,41,42].

Another interesting finding was that CIMP-low tumors had a significantly higher mutation rate in *PIK3CA* than CIMP-high and CIMP-0 tumors. Nearly 50% of CIMP-low tumors had *PIK3CA* mutations. These findings were in contrast to those from previous studies [35,43] that showed that the CIMP-high status was significantly associated with the *PIK3CA* mutation. However, the frequency of *PIK3CA* mutations in CIMP-high tumors was approximately 20% in these studies and similar to the frequency of *PIK3CA* mutations in CIMP-high (21.2%) tumors in our series. Another possible explanation for this difference is the level of the CIMP. As shown in Figure 1, most tumors with *PIK3CA* mutations had at least three hypermethylation markers. Therefore, *PIK3CA* mutations might overlap with aberrant DNA methylation pathways. 

In contrast, MSI-high and CIMP-high CRCs had a significantly higher frequency of mutations in *BRAF*, *SMAD4*, *MSH3*, and *POLD1* than CIMP-0 and CIMP-low tumors. Despite its crucial role in cellular function, the dysregulation of CpG methylation is considered mutagenic. Ehrlich’s study found that methylated cytosines more easily undergo spontaneous deamination than unmethylated cytosines, leading to a cytosine to thymine mismatch in DNA synthesis [44]. Poulos’s study analyzed tissue-specific methylation data including the TCGA and International Cancer Genome Consortium (ICGC) data. They found a strong association between C>T mutations and methylation at CpG dinucleotides in many cancer types [45]. Furthermore, their results and those from other studies also suggested that MMR proteins are partly involved in the correction of C>T mutations [45,46]. However, the difference between mutations in specific genes and different CIMP statuses still deserves attention.

Patients with sporadic MSI-high and CIMP-positive CRCs were older in age and showed a proximal colon predominance and a mucinous histology [12,13,14,15]. Over time, the DNA methylation pattern becomes altered, especially global hypomethylation and hypermethylation in specific CpG islands [22,23,24]. Similar to other studies, we also found that MSI-high CRCs with the CIMP were located predominantly in the proximal colon. One possible explanation for this observation is that the carcinogen in the proximal colon affects DNA methylation, resulting in cytosine to thymine mutations, as mentioned in a previous study [41]. Deficient MMR results in the accumulation of mutations and CRC carcinogenesis. Further mutations in specific genes, including *SMAD4* and *BRAF*, alter the *TGF-beta* and *MAPK* signaling pathways. In vitro and clinical observation studies have reported associations between differentiation, mucinous histology, and altered *TGF-beta* and *MAPK* signaling pathways [47,48,49].

In contrast to other studies’ conclusions that the prognosis of CRC patients with the CIMP is poor [14,15], our results showed that patients with sporadic MSI-high and CIMP-high CRC tended to have higher DFS rates than those with CIMP-low or CIMP-0 CRC, although the difference was not statistically significant. This finding may be explained by the fact that slightly more patients with CIMP-low or CIMP-0 tumors were in stage IV than those with CIMP-high tumors (15.9% vs. 8.7%). In the multivariate analysis, the prognoses of CIMP-positive and CIMP-negative patients were similar.

## 4. Materials and Methods

After obtaining approval from the Institutional Review Board of Taipei Veterans General Hospital (2017-12-011CC), we obtained information on 92 patients who were diagnosed with MSI-high CRC from a database consisting of 1505 patients with CRC who received surgery at the hospital between 2000 and 2010. These 92 patients were confirmed to have no germline mutation in 5 major MMR genes (*EPCAM*, *MLH1*, *MSH2*, *MSH6*, and *PMS2*) by NGS and Sanger sequencing. This database prospectively collected clinical information including age, sex, personal and family medical histories, tumor location, and carcinoembryonic antigen level and pathological data including tumor, node, metastasis (TNM) stage, differentiation, mucinous histology, inflammation in the stroma, and lymphovascular invasion (LVI). Before data collection, informed consent was signed before the patients received surgery. This database did not include patients who died within 30 days of surgery, received preoperative chemoradiotherapy, or underwent emergent operations. The follow-up timeline was described as follows: patients were monitored every 3 months for the first 2 years and semiannually thereafter. The follow-up protocol included a physical examination, digital rectal examination, carcinoembryonic antigen analysis, chest radiography, abdominal sonogram, and computerized tomography, if needed. Proton emission tomography or magnetic resonance imaging was arranged for patients with elevated levels of carcinoembryonic antigen but an unknown site of tumor recurrence.

### 4.1. MSI Analysis

According to international criteria [4], the following five reference microsatellite markers were used to determine the MSI phenotype: D5S345, D2S123, BAT25, BAT26, and D17S250. DNA from the tumor and corresponding normal mucosa were amplified with polymerase chain reaction (PCR) [50,51]. The PCR products were genotyped on an ABI 3700 automated sequencer (Applied Biosystems, Foster City, CA, USA). Samples with ≥2 positive MSI markers were defined as MSI-high, and those with 0–1 positive MSI markers were defined as MSS.

### 4.2. Targeted NGS

We took advantage of the high-throughput Illumina HiSeq2500 system (Illumina Inc., San Diego, CA, USA) to comprehensively explore the DNA sequences of all exons of 5 well-known MMR genes (*EPCAM*, *MLH1*, *MSH2*, *MSH6*, and *PMS2*) and another 17 common CRC-associated genes (*APC*, *AXIN1*, *AXIN2*, *BAX*, *BRAF*, *CTNNB1*, *EXO1*, *FBXW7*, *KRAS*, *NRAS*, *MSH3*, *PIK3CA*, *PMS1*, *POLD1*, *POLE*, *SMAD4*, and *TP53*) (selected from a previous study and the COSMIC database) [40,52]. A total of 100 ng of DNA from each individual was used to construct a sample library using the Roche KAPA Library Preparation Kit (KAPA Biosystems, Wilmington, MA, USA). Each DNA was fragmented and then used to prepare the DNA library by performing end-repair, a-overhang addition, adaptor ligation, and size selection (150~350 bp). The target DNAs of the DNA repair-related genes were enriched using probe-based methods. The probes were synthesized by Integrated DNA Technologies (Coralville, IA, USA) according to our previously designed probe sequences, and the capture procedure was performed following IDT guidelines. After probe-based enrichment, the library of each pool was amplified with 14 cycles. The amplified libraries were quantified using a qPCR system and pooled into a new 1.5 mL tube as a 10nM pooled DNA library. The final pool was used for sequencing (Illumina HiSeq2500 sequencer, 2 × 100 bp). The raw output of each individual was 250 Mb, and the average depth of the target region was >1000X. The sequence of each read was trimmed based on the quality score (Q30), and the length of each read less than 45 bp was discarded in the subsequent analysis. Reads were aligned to the human hg19 reference genome using BWA-MEM (http://bio-bwa.sourceforge.net/), and GATK Unifiedgenotyper program (GATK Lite version 2.3–9; https://gatk.broadinstitute.org/hc/en-us) [53] was used to call variants. After variant calling, we used Variant Effect Predictor (http://grch37.ensembl.org/Homo_sapiens/Tools/VEP) [54] to annotate the identified variants for the subsequent statistical analysis. The mean read depths were 2404.2 ± 1457.7 and 4119.4 ± 2991.1 in the germline and somatic mutation analyses, respectively.

### 4.3. Sanger Sequencing

To detect germline mutations in *EPCAM*, *MLH1*, *MSH2*, *MSH6*, and *PMS2*, DNA obtained from the blood was amplified and sequenced with primers used in previous studies [55,56,57]. Sequencing of these five MMR genes covered their exons and the intronic regions adjacent to all splice sites. Briefly, the extracted DNA was amplified selectively by PCR in a DNA thermocycler. A negative control containing no DNA template was included for each PCR amplification round. The PCR products were analyzed on an automated sequencer (ABI Prism 3100 Genetic Analyzer; Perkin Elmer Applied Biosystems, Waltham, MA, USA). Each sample was sequenced on both the sense and antisense strands. Each mutation was confirmed by a second sequencing procedure on new PCR products. By comparing the obtained sequence with the known sequence, nonsense, missense, and frameshift mutations were identified. Nonsense and frameshift mutations were considered pathogenic. Because missense changes in MMR genes do not necessarily affect the function of the protein, we considered a missense mutation in an MMR gene without abnormal protein expression [58,59].

### 4.4. CIMP Analysis

DNA samples of tumor tissues were bisulfite-converted using the EpiTect Fast Bisulfite Conversion Kit (Qiagen, Valencia, CA, USA). The converted DNA samples were analyzed for their methylation status of eight CIMP target genes (*CACNA1G*, *CDKN2A* (*P16*), *CRABP1*, *IGF2*, *MLH1*, *NEUROG1*, *RUNX3*, and *SOCS1*) and ALU markers using methylation-specific real-time quantitative PCR technology. The DNA methylation status of each examined marker was quantified and is reported as the percent of the methylated reference (PMR), which was proposed in a previous study [4,60]. The PMR represents 100 × ((methylated reaction/ALU) sample/(methylated reaction/ALU) reference). A CpG island locus was considered methylated when an exponential amplification curve was present and the PMR value was >4. Each tumor was classified as CIMP-high, CIMP-low, or CIMP-0 if it exhibited methylation of >4 markers, 1–4 markers, or no marker, respectively.

### 4.5. Statistical Analysis 

The statistical endpoint for disease-free survival (DFS) and overall survival (OS) was measured from the date of surgery until the date the patient experienced tumor recurrence or died from any cause. Patients with an unknown survival status were censored on the date of their last follow-up. Kaplan–Meier survival curves were plotted and compared using the log-rank test. The impact of the molecular and clinicopathological features on OS was assessed using univariate analysis. The covariates significantly associated with OS by univariate analysis were included in the multivariate Cox regression analysis. Chi-squared and two-tailed Fisher’s exact tests were used to compare the genotype frequency of clinicopathological features. Numerical values were compared using Student’s t-test. Data are expressed as the mean ± standard deviation. Statistical significance was defined as *p* < 0.05. Statistical analyses were performed using IBM SPSS Statistics 25.0 (IBM Corp., Armonk, NY, USA).

## 5. Conclusions

Only TNM stage was a statistically significant predictor of outcomes independent of CIMP profiles in MSI-high CRC patients. Sporadic MSI-high CRCs with different mechanisms of carcinogenesis have specific mutation profiles and clinicopathological features.

## Figures and Tables

**Table 1:** Clinicopathological features of microsatellite instability (MSI)-high colorectal cancers (CRC) classified by 5’-C-phosphate-G-3’ island methylator phenotype (CIMP) status.

Variables	Total Patients *n* = 92 *n* (%)	CIMP-0 *n* = 25 *n* (%)	CIMP-Low *n* = 44 *n* (%)	CIMP-High *n* = 23 *n* (%)	*p* Value
Age	69.0 ± 14.6	68.0 ± 15.2	68.4 ± 15.7	71.3 ± 12.1	0.690
Gender					
Male	49 (53.2)	18 (72.0)	25 (56.8)	6 (26.1)	**0.005**
Female	43 (57.6)	7 (28.0)	19 (43.2)	17 (73.9)	
Location					
Proximal colon	45 (48.9)	9 (36.0)	17 (38.6)	19 (82.6)	**0.002**
Distal colon	20 (21.7)	7 (28.0)	9 (20.5)	4 (17.4)	
Rectum	27 (29.3)	9 (36.0)	8 (40.9)	0	
TNM stage					
I	11 (12.0)	4 (16.0)	4 (9.1)	3 (13.0)	0.415
II	48 (52.2)	15 (60.0)	20 (45.5)	13 (56.5)	
III	20 (21.7)	5 (20.0)	10 (22.7)	5 (21.7)	
IV	13 (14.1)	1 (4.0)	10 (22.7)	2 (8.7)	
Lymphovascular invasion					
Positive	14 (15.2)	5 (20.0)	6 (13.6)	3 (13.0)	0.736
Negative	78 (8.7)	20 (80.0)	38 (86.4)	20 (87.0)	
Cell Differentiation					
Well/moderate	77 (83.7)	23 (92.0)	39 (88.6)	15 (65.2)	**0.020**
Poor	15 (16.3)	2 (8.0)	5 (11.4)	8 (34.8)	
Mucinous component					
≥50%	13 (14.1)	3 (12.0)	5 (11.4)	5 (21.7)	0.480
<50%	79 (85.9)	22 (88.0)	39 (88.6)	18 (78.3)	

CIMP: 5’-C-phosphate-G-3’ islands methylation phenotype; TNM: tumor, node, metastasis; bold: statistically significant.

**Table 2:** Molecular signatures of MSI-high CRC classified by CIMP status.

Genetic Mutation	Total Patients *n* = 92 *n* (%)	CIMP-0 *n* = 25 *n* (%)	CIMP-Low *n* = 44 *n* (%)	CIMP-High *n* = 23 *n* (%)	*p* Value
*APC*	40 (43.4)	25 (100)	12 (27.3)	3 (13.0)	**<0.001**
*TP53*	41 (44.6)	25 (100)	14 (31.8)	2 (8.7)	**<0.001**
*KRAS*	45 (48.9)	15 (60.0)	26 (59.1)	4 (17.4)	**0.001**
*PIK3CA*	33 (35.9)	3 (12.0)	23 (52.3)	7 (30.4)	**0.003**
*FBXW7*	24 (26.1)	2 (8.0)	19 (43.2)	3 (13.0)	**0.002**
*NRAS*	6 (7.5)	4 (16.0)	2 (4.5)	0	0.062
*CTNB1*	13 (14.1)	4 (16.0)	3 (6.8)	6 (26.1)	0.094
*BAX*	48 (52.2)	13 (52.0)	19 (43.2)	16 (69.6)	0.122
*AXIN1*	10 (10.9)	1 (4.0)	4 (9.1)	5 (21.7)	0.125
*AXIN2*	28 (30.4)	6 (24.0)	15 (34.1)	7 (30.4)	0.692
*EXO1*	12 (13.0)	2 (8.0)	5 (11.4)	5 (21.7)	0.332
*POLE*	44 (47.8)	11 (44.0)	23 (52.3)	10 (43.5)	0.715
*PMS1*	76 (82.7)	19 (76.0)	36 (81.8)	21 (91.3)	0.370
*BRAF*	21 (22.8)	3 (12.0)	4 (9.1)	14 (60.9)	**<0.001**
*SMAD4*	15 (16.3)	1 (4.0)	7 (15.9)	7 (30.4)	**0.046**
*MSH3*	24 (26.1)	6 (24.0)	6 (13.6)	12 (52.2)	**0.003**
*POLD1*	36 (39.1)	6 (24.0)	16 (36.4)	14 (60.9)	**0.029**

CIMP: 5’-C-phosphate-G-3’ islands methylation phenotype; bold: statistically significant.

**Table 3:** The clinicopathological features between CRC patients with and without mismatch repair (MMR) mutation.

Variables	MMR Mutation (+) *n* = 64 *n* (%)	MMR Mutation (−) *n* = 28 *n* (%)	*p* Value
Age (year)	69.3 ± 14.9	68.4 ± 14.2	0.804
Gender			
Male	36 (56.3)	13 (46.4)	0.496
Female	28 (43.8)	15 (53.6)	
Location			
Proximal colon	33 (51.6)	12 (42.4)	0.739
Distal colon	13 (20.3)	7 (20.3)	
Rectum	18 (28.1)	9 (32.1)	
TNM stage			
I	7 (10.9)	4 (14.3)	0.365
II	37 (57.8)	11 (39.3)	
III	13 (20.3)	7 (25.0)	
IV	7 (10.9)	6 (21.4)	
Lymphovascular invasion			
Positive	7 (10.9)	7 (25.0)	0.115
Negative	57 (89.1)	21 (75.0)	
Cell Differentiation			
Well/moderate	54 (84.4)	23 (82.1)	0.768
Poor	10 (15.6)	5 (17.9)	
Mucinous component			
≥50%	13 (20.3)	0	**0.008**
<50%	51 (79.7)	28 (100)	

TNM: tumor, node, metastasis; MMR: mismatch repair; bold: statistically significant.

**Table 4:** The molecular signatures of MSI-high CRC classified by somatic MMR mutations.

Genetic Mutation	MMR Mutation (+) *n* = 64 *n* (%)	MMR Mutation (−) *n* = 28 *n* (%)	*p* Value
*APC*	24 (37.5)	16 (57.1)	0.110
*TP53*	28 (43.8)	17 (60.7)	0.265
*KRAS*	41 (59.3)	4 (17.4)	0.175
*BRAF*	11 (17.2)	10 (35.7)	0.063
*SMAD4*	11 (17.2)	4 (14.3)	1.000
*MSH3*	19 (29.7)	5 (17.9)	0.306
*POLD1*	30 (46.9)	6 (21.4)	**0.036**
*CTNB1*	11 (1.2)	2 (7.1)	0.330
*BAX*	36 (56.3)	12 (42.9)	0.254
*AXIN1*	9 (14.2)	1 (3.6)	0.273
*AXIN2*	22 (34.4)	6 (21.4)	0.325
*FBXW7*	14 (21.9)	10 (35.7)	0.200
*EXO1*	10 (15.6)	2 (7.1)	0.333
*NRAS*	4 (6.3)	2 (7.1)	1.000
*POLE*	31 (48.4)	13 (46.4)	1.000
*PIK3CA*	26 (40.6)	7 (25.0)	0.166
*TGFBR2*	64 (100)	28 (100)	1.000
*PMS1*	53 (82.8)	23 (82.1)	1.000

MMR: mismatch repair; bold: statistically significant.

**Table 5:** Univariate and multivariate analysis of factors affecting overall survival (OS) of CRC patients.

Factors	Univariate Analysis			Multivariate Analysis		
	Hazard Ratio	95% CI	*p* Value	Hazard Ratio	95% CI	*p* Value
TNM stage	3.39	2.17–5.30	**<0.001**	4.22	2.245–7.923	**<0.001**
LVI	3.49	1.54–7.91	**0.003**	2.30	0.927–5.691	0.073
Mucinous histology	0.44	0.13–1.47	0.182			
Poor differentiation	1.98	0.85–4.61	0.115			
CIMP status	0.99	0.44–2.22	0.982			
Somatic MMR mutation	0.69	0.33–1.44	0.320			
Adjuvant chemotherapy	2.80	1.36–5.76	**0.005**	0.49	0.166–1.445	0.196

CI: confidence interval; CIMP: CpG islands methylation phenotype; MMR: mismatch repair; LVI: lymphovascular invasion; bold: statistically significant

## Supplementary Materials

The following are available online at https://www.mdpi.com/2072-6694/12/11/3487/s1, Table S1: The MMR immunohistochemical status for each protein, methylation status for each CIMP marker, and mutation status of MMR genes stratified by CIMP status in MSI-H CRC.

## Abbreviations

CRC	Colorectal Cancer
MSI	Microsatellite Instability
MSS	Microsatellite Stable
MMR	Mismatch Repair
LS	Lynch syndrome
CIMP	CpG Island Methylation Phenotype
NGS	Next-Generation Sequencing
LVI	Lymphovascular Invasion
PCR	Polymerase Chain Reaction
PMR	Percent Of The Methylated Reference
OS	Overall Survival
DFS	Disease-Free Survival
TNM	Tumor, Node, Metastasis
TCGA	The Cancer Genome Atlas
ICGC	International Cancer Genome Consortium
