# Supplementary material for: Clinicopathological and Molecular Profiles of Sporadic Microsatellite Unstable Colorectal Cancer with or without the CpG Island Methylator Phenotype (CIMP)
Source: Cancers (Basel). 2020 Nov 23;12(11):3487. doi: 10.3390/cancers12113487 (PMC7700556; doi:10.3390/cancers12113487)
Supplement: Supplementary file 1 [file cancers-12-03487-s001.pdf]

SupplementaryMaterials

Clinicopathological and Molecular Profiles of Sporadic Microsatellite Unstable Colorectal Cancer with or without the CpG Island Methylator Phenotype (CIMP)

Shih-Ching Chang, Anna Fen-Yau Li, Pei-Ching Lin, Chun-Chi Lin, Hung-Hsin Lin, Shen-Chieh Huang, Chien-Hsing Lin, Wen-Yi Liang, Wei-Shone Chen, Jeng-Kai Jiang, Jen-Kou Lin, Shung-Haur Yang and Yuan-Tzu Lan

Table S1. The MMR immunohistochemical status for each protein, methylation status for each CIMP marker and mutation status of MMR genes stratified by CIMP status in MSI-H CRC.

	**Total Patients n = 92**	**CIMP-0 n = 25**	**CIMP-Low n = 44**	**CIMP-high n = 23**	***p* value**
Loss of MMR Protein Expression	42 (45.7)	8 (32.0)	17 (38.6)	17 (73.9)	**0.006**
MSH2	5 (5.4)	1 (4.0)	2 (4.5)	2 (8.7)	0.482
MSH6	8 (8.7)	3 (12.0)	0	5 (21.7)	0.268
MLH1	26 (28.3)	3 (12.0)	10 (22.7)	13 (56.5)	**0.002**
PMS2	37 (40.2)	6 (24.0)	17 (38.6)	14 (60.9)	**0.032**
Methylation Status of CIMP Marker					
CACNA1G	36 (39.1)	0	13 (29.5)	23 (100)	**<0.001**
CDKN2A (P16)	39 (42.4)	0	25 (56.8)	14 (60.9)	**<0.001**
CRABP1	44 (47.8)	0	21 (47.4)	23 (100)	**<0.001**
IGF2	30 (32.6)	0	13 (29.5)	17 (73.9)	**<0.001**
MLH1	24 (26.1)	0	3 (6.8)	21 (91.3)	**<0.001**
NEUROG1	17 (18.5)	0	4 (9.1)	13 (56.5)	**<0.001**
RUNX3	20 (87.0)	0	10 (22.7)	20 (87.0)	**<0.001**
SOCS1	15 (16.3)	0	5 (11.4)	10 (43.5)	**<0.001**
MMR Mutation	64 (69.6)	16 (64.0)	31 (70.5)	17 (73.9)	0.745
MSH6	40 (43.5)	8 (32.0)	19 (43.2)	13 (56.5)	0.231
MLH1	20 (21.7)	5 (20.0)	10 (22.7)	5 (21.7)	0.966
MSH2	16 (17.4)	6 (24.0)	5 (11.4)	5 (21.7)	0.337
PMS2	19 (20.7)	5 (20.0)	10 (22.7)	4 (17.4)	0.873
EPCAM	6 (6.5)	2 (8.0)	3 (6.8)	1 (4.3)	0.872

CIMP: 5′-C-phosphate-G-3′ island methylator phenotype; MMR: mismatch repair; bold: statistically significant.
